# Applying muscle synergy analysis to forearm high-density electromyography of healthy people

**DOI:** 10.3389/fnins.2022.1067925

**Published:** 2022-12-20

**Authors:** Yanjuan Geng, Ziyin Chen, Yang Zhao, Vincent C. K. Cheung, Guanglin Li

**Affiliations:** ^1^Shenzhen Institute of Advanced Technology, Chinese Academy of Sciences, Shenzhen, China; ^2^School of Biomedical Sciences, The Gerald Choa Neuroscience Institute, The Chinese University of Hong Kong, Hong Kong, Hong Kong SAR, China

**Keywords:** high-density electromyography, muscle synergy, isometric, isotonic, wrist motion

## Abstract

**Introduction:**

Muscle synergy is regarded as a motor control strategy deployed by the central nervous system (CNS). Clarifying the modulation of muscle synergies under different strength training modes is important for the rehabilitation of motor-impaired patients.

**Methods:**

To represent the subtle variation of neuromuscular activities from the smaller forearm muscles during wrist motion, we proposed to apply muscle synergy analysis to preprocessed high-density electromyographic data (HDEMG). Here, modulation of muscle synergies within and across the isometric and isotonic training modes for strengthening muscles across the wrist were investigated. Surface HDEMGs were recorded from healthy subjects (*N* = 10). Three different HDEMG electrode configurations were used for comparison and validation of the extracted muscle synergies. The cosine of principal angles (CPA) and the Euclidian distance (ED) between synergy vectors were used to evaluate the intra- and inter-mode similarity of muscle synergies. Then, how the activation coefficients modulate the excitation of specific synergy under each mode was examined by pattern recognition. Next, for a closer look at the mode-specific synergies and the synergies shared by the two training modes, *k*-means clustering was applied.

**Results:**

We observed high similarity of muscle synergies across different tasks within each training mode, but decreased similarity of muscle synergies across different training modes. Both intra- and intermode similarity of muscle synergies were consistently robust to electrode configurations regardless of the similarity metric used.

**Discussion:**

Overall, our findings suggest that applying muscle synergy analysis to HDEMG is feasible, and that the traditional muscle synergies defined by whole-muscle components may be broadened to include sub-muscle components represented by the HDEMG channels. This work may lead to an appropriate neuromuscular analysis method for motor function evaluation in clinical settings and provide valuable insights for the prescription of rehabilitation training therapies.

## 1 Introduction

Upper limb muscle weakness is a common impairment after central nervous system (CNS) diseases or injury (Dalise et al., 2020). Strength training has been suggested as one of the therapies to restore strength in the paretic arm of patients in several clinical trials (Harris and Eng, 2010). Among variable contraction type of exercises that applied for strength training, isometric and isotonic modes are commonly used in clinical settings (Guilhem et al., 2010; Remaud et al., 2010). Although these training modes are both dynamic in nature, they are different at the level of muscle. Specifically, isometric exercise involves muscle contraction without moving of muscle, while during an isotonic contraction the muscle tension varies throughout the range of motion. Therefore, to clarify the characteristics of muscle contraction under different training modes becomes essentially important, particularly for physiotherapist who need to prescribe appropriate therapies for individuals with different levels of motor impairment.

Using simple metrics from selected muscle pairs, such as the sEMG-based co-contraction index, fuzzy approximate entropy, and power spectral analysis (Guanglin Li et al., 2014; Sin et al., 2014; Sun et al., 2014), different researchers have attempted to describe the neuromuscular adaptions to variant exercises in previous studies. For a closer look at the pathological alteration, some researchers extracted motor unit firing rates, recruitment thresholds, and action potential amplitudes through decomposition of the high-density sEMG signals to evaluate the altered motor unit firing behaviors (Li et al., 2011, 2015; Hu et al., 2016; Dai et al., 2017; Murphy et al., 2018). Note that since these studies involved only a pair of agonist and antagonist muscles or a specific muscle, they could not characterize the coordinated muscular activities of related muscle group. The muscle synergy analysis, which describes patterns of multiple coordinated muscles, might be a more appropriate method. It is regarded as a motor control strategy by the CNS. Specifically, the generated motion is driven by a linear combination of muscle synergies, each of which activates a group of muscles as a single unit.

Despite that the physiological origin and operational mechanism of muscle synergies are still being dissected (Cheung and Seki, 2021), an increasing number of studies have utilized muscle coordination to quantify the impairment level after CNS diseases or injury (Li et al., 2017; Tang et al., 2017; Shuman et al., 2019; Rinaldi et al., 2020; Kubota et al., 2021), or to evaluate the effect of physical training based on the variation of muscle synergy (Kogami et al., 2018; Hu et al., 2019; Yang et al., 2020). More recently, muscle synergy was also used to investigate the modulation mechanism of motor learning and motor adaptions (Jacobs et al., 2018; Yang et al., 2019; Cheung et al., 2020a,b; Rinaldi et al., 2020; Chang and Agrawal, 2022). For example, Cheung et al., 2020a,b found that the variability modulation of specific muscle synergies and their activations contribute to early motor learning, and further revealed the possible mechanism for modifying early motor modules to accommodate the changing limb biomechanics and influences from sensorimotor training. This literature suggests that muscle synergy analysis may provide valuable insights on the characteristics of muscle contraction under different training modes. It is noteworthy, however, that 10 or so sEMG channels were commonly utilized in these studies, and the placement of sEMG electrodes mainly relies on the anatomical experience of the experimenter, which may lead to suboptimal positioning as a result. Particularly, for a fine movement that requires coordination of many small muscles underlying the forearm, sparsely placed electrodes might not be able to capture subtle variation of neuromuscular activities.

Therefore, aiming at clarifying the modulation of muscle synergies under isometric and isotonic wrist movements, a high-density EMG (HDEMG) based muscle synergy analyzing method was proposed in this work. The rationale is that with HDEMG, there is a better chance that activities of individual muscles can be picked up by a single sensor. Moreover, HDEMG permits more spatial resolution in the recording of muscle activities, so that it may even be possible to isolate activities of different within-muscle compartments that are under differential control of the CNS. In this work, the feasibility of applying muscle synergy analysis to HDEMG was first validated. We hypothesized that the intra-mode muscle synergy patterns are similar across different force levels or weights, while the inter-mode similarity of muscle synergies would be smaller for each movement. In other words, different physical training modes may affect the modulation of muscle synergies for the same movement, because the muscle activation would change according to the definition of isometric contraction and isotonic contraction. If so, these possible reasons account for the need to investigate our scientific problem further.

## 2 Methods

### 2.1 Participants and experiment

In this study, we recruited 10 subjects without any history of neurological or neuromuscular impairments (age: 24.5 ± 1.5 years, 7 males and 3 females). All participants gave written informed consent and provided permission for publication of their photographs and data for scientific and educational purposes. The experimental protocol of this study was approved by the Research Ethics Board of Shenzhen Institute of Advanced Technology, Chinese Academy of Sciences.

**FIGURE 1:**
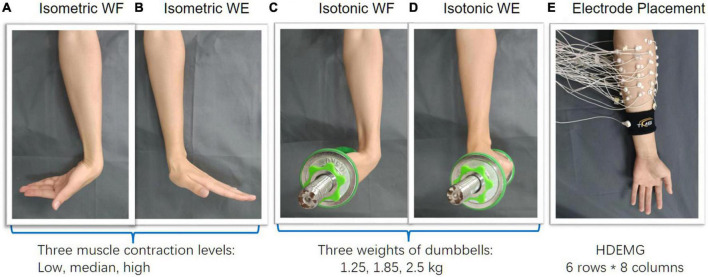
Experimental protocol. **(A–D)** Wrist flexion/extension were separately performed under isometric and isotonic mode, **(E)** 48-channel HDEMG was recorded meanwhile.

In the experiment, each participant was asked to perform wrist flexion (WF) and wrist extension (WE) under isometric mode and isotonic mode with their right hands, respectively. To depict the muscle coordination more comprehensively, three different muscle contraction force levels, i.e., low (L), median (M), and high (H) which were about 30, 50, 70% of maximum of voluntary contraction (MVC) force were included under the isometric mode (Figures 1A, B). In this study, the MVC force to perform WE and WF was measured with an ergometer before each session for each subject. Visual feedback based on the maximum EMG amplitude was provided to the subjects to regulate the muscle contraction force. With a constant muscle contraction force, each movement was maintained for 4 s and repeated 10 times by following the sound from a metronome, which gave audio reminder at the beginning and the end of each motion repetition. A rest time of 3 s was set between two successive movements in each trial to avoid muscle fatigue. To perform isotonic movement with variant muscle-tension, three different weights of dumbbells were utilized (Figures 1C, D). Each subject was required to perform wrist flexion and then wrist extension in the sagittal plane with a constant speed of 4s/round. Both wrist flexion and wrist extension were repeated 10 times.

During wrist movements, a HDEMG acquisition system (Refa-128, TMS International BV, Netherlands) was utilized for data recording. A total of 48 monopolar electrodes (5 mm in diameter) were placed on the forearm circumference in an 8 × 6 grid from 1 cm proximal to the elbow crease to 1/3 distal to the wrist joint with an electrode inter-distance (EID) of around 2 cm. The forearm muscles we aimed to cover with are extensor carpi radialis longus, extensor carpi radialis brevis, flexor carpi ulnaris, extensor carpi ulnaris, extensor digiti minimi, extensor digitorum, abductor pollicis longus, as well as some muscles underlying these superficial forearm muscles. Considering different forearm sizes for different subjects, we put a plastic string which has eight evenly distributed markers on the forearm circumference, proximal to the elbow crease at first. Then scroll the plastic string down to 1/3 distal to the wrist joint along the vertical direction. In this way, the placement of the 48 monopolar electrodes were marked beforehand (Figure 1E). A reference electrode was fixed on a nylon bracelet that was worn on subject’s wrist. The sampling rate of EMG signals was set as 1,024 Hz.

### 2.2 Data preprocessing and electrode configuration

The recorded HDEMG for each movement under each training mode were band-pass filtered (4th order Butterworth filter, 30–450 Hz), rectified, and low-pass filtered (4th order Butterworth filter, 20 Hz) to obtain the envelope of the EMG signals. Then, the EMG envelopes from 500 ms after the onset of movement to 200 ms before the offset of the movement was selected and concatenated for each trial.

**FIGURE 2:**
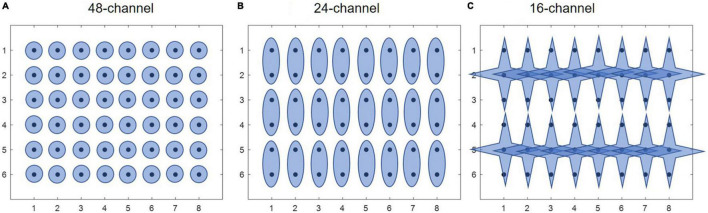
Three different electrode configurations. **(A)** 48-channel, **(B)** 24-channel, and **(C)** 16-channel.

Afterward, three different electrode configurations were formed for subsequent muscle synergy analysis. The main purpose of including different electrode configurations is to validate the robustness of HDEMG-based muscle synergy by mean of comparison. Herein, the first configuration was composed of 48 monopolar electrodes (Figure 2A), the second configuration was composed of 24 differential channels by subtracting each pair of adjacent electrodes along the muscular fibers (Figure 2B), and the third configuration was computed by introducing a Laplace operator (Figure 2C). For each movement under each training mode, three HDEMG matrixes corresponding to three electrode configurations were used for subsequent muscle synergy analysis.

### 2.3 Extraction of muscle synergies from HDEMG

For each preprocessed HDEMG matrix X, a non-negative matrix factorization (NMF) algorithm, named alternating non-negativity constrained least squares and active set (NNLS) was used to factorize X into a linear combination of muscle synergy vectors W, and temporal activation patterns H (Eq. 1).


(1) $$\begin{array}{c} X^{M*T}\approx W^{M*N}*H^{N*T} \end{array}$$

where X is the HDEMG dataset, M denotes the number of muscles, T denotes the number of time samples, W is the synergies matrix (M*N, where N is the number of synergies), and H is the coefficient matrix. Each column of W represents the weights of each muscle for a single synergy, and each row of H represents the activation profile for the corresponding synergy.

Herein, we adopted the NNLS algorithm because it outputs the unit-vector synergies and is fast to convergence to a stationary point (Li and Ngom, 2013; Yanjuan Geng et al., 2020). For each input X, the NNLS algorithm was executed 20 times for a range of 1–M possible number of muscle synergies. For every possible number of muscle synergies *N*, the set of muscle synergies extracted from these 20 iterations with the highest variance accounted for (VAF) the observed signal X was retained. The definition of VAF was shown as follows:


(2) $$\begin{array}{c} V\,A\,F=\frac{{||X-W*H||}^{2}}{{||X-m\,e\,a\,n\,\left(X\right)||}^{2}} \end{array}$$

To estimate the optimal number of muscle synergies, a regression-based searching method was used (Geng et al., 2020). First, a curve plot of VAF against N was plotted, where N denotes the number of extracted synergies ranging from 1 to M (corresponding to M muscles). Then, a series of linear regression procedures were performed to examine which VAF vs. N curve was essentially straight. Reference (Geng et al., 2020) provided more details about this step.

### 2.4 Evaluating the structure similarity of muscle synergies

The main objective of this study is to evaluate the intra-mode and inter-mode similarity of muscle synergies, respectively. Firstly, the structure similarity of muscle synergies across three different force levels (or weights) was computed under isometric mode (or isotonic mode), then the inter-mode analysis was performed in order to Figure out the similarity of muscle synergies between isometric and isotonic exercises. To quantify the structure similarity of muscle synergies, two similarity metrics, i.e., the Cosine of principal angles (CPA) and the Euclidian distance (ED) were used, which were defined as follows:


(3) $$\begin{array}{c} C\,P\,A\,\left(W_{p}^{a},W_{q}^{b}\right)=\frac{\sum_{i=1}^{p}\begin{array}{c} q\\ m\,a\,x\\ j=1 \end{array}\,\left(\frac{w_{i}^{a}}{{||w_{i}^{a}||}_{2}}*\frac{w_{j}^{b}}{{||w_{j}^{b}||}_{2}}\right)}{2\,p}+\\ \frac{\sum_{j=1}^{q}\begin{array}{c} p\\ m\,a\,x\\ i=1 \end{array}\,\left(\frac{w_{i}^{a}}{{||w_{i}^{a}||}_{2}}*\frac{w_{j}^{b}}{{||w_{j}^{b}||}_{2}}\right)}{2\,q} \end{array}$$

(4) $$\begin{array}{c} E\,D\,\left(W_{p}^{a},W_{q}^{b}\right)=\frac{\sum_{i=1}^{p}\begin{array}{c} q\\ m\,i\,n\\ j=1 \end{array}\,\left({||w_{i}^{a}-w_{j}^{b}||}_{2}\right)}{2\,p}+\\ \frac{\sum_{j=1}^{q}\begin{array}{c} p\\ m\,i\,n\\ i=1 \end{array}\,\left({||w_{i}^{a}-w_{j}^{b}||}_{2}\right)}{2\,q} \end{array}$$

where $w_{i}^{a}$ is the *i*th synergy of matrix $W_{p}^{a}$, $w_{j}^{b}$ is the *j*th synergy of $W_{q}^{b}$, *p* and *q* are the number of synergies in $W_{p}^{a}$ and $W_{q}^{b}$, respectively. Note each synergy obtained by implementing the NNLS algorithm was a unit vector, so the maximum similarity value was one when using CPA, and the minimum similarity value was zero when using ED. The higher the CPA value is, or the lower of ED values is, the more similar the paired muscle synergies would be. To indicate the significance of similarity under each condition, a CPA threshold which was defined as the 80th percentile of the CPAs from all possible paired synergy vectors between any two force levels (weights, or exercise modes) was computed. Similarly, an ED threshold was computed as the 20th percentile of EDs from all possible paired synergy vectors between any two force levels (weights, or exercise modes).

### 2.5 Quantification of activation coefficients

If the structure of muscle synergies is similar, how the activation coefficients modulate the excitation of specific synergy needs to be investigated in further. In this work, the activation coefficients corresponding to different muscle contraction force levels (and weights) were classified by means of pattern recognition, and the classification accuracy (CA) was used as the metric to evaluate the separability of activation coefficients among different force levels (and weights). Given the optimal number of muscle synergies varied among different force levels for a specific movement, the number of activation coefficient vectors varied accordingly, we firstly reduced the dimension of activation coefficients matrices using principal component analysis. Then a sliding window with length of 100 data points and increment of 100 data points was used for window segmentation. From each window the mean value was extracted to form the feature matrix, which was then fed into the linear discriminant analysis (LDA) classifier for pattern recognition.

### 2.6 Extracting shared synergies and mode-specific synergies

In this study, we hypothesized that for each movement, the inter-mode similarity of muscle synergies would reduce in comparison to the intra-mode similarity. If so, the possible reasons that affect the similarity attenuation need to be investigated. For this purpose, for each motion under each exercise mode (i.e., isometric WF, isometric WE, isotonic WF, and isotonic WE), all muscle synergy vectors from all subjects were clustered by using K-means clustering algorithm. Afterward, muscle synergies shared by isometric mode and isotonic mode, synergies specific to each training mode were computed in terms of the CPA.

For synergy clustering, the optimal number of clusters need to be determined. Herein we adopted the elbow method to evaluate how good a clustering outcome is for various values of K and find the elbow point, i.e., the optimal number of clusters. Initially, the quality of clustering improves rapidly when changing the value of K but eventually stabilizes. The elbow point was the point where the relative improvement was not very high anymore based on the ratio of within-cluster mean distance to inter-cluster mean distance. Next, clusters that were not representative and subject-invariant were deleted from the obtained clusters. The criterion was that the number of muscle synergies in these clusters was less than 1/3 of the value, i.e., the total number of muscle synergies from all subjects divided by the optimal number of clusters. Subsequently, shared muscle synergies and mode-specific synergies were computed in term of the similarity of cluster centroids. In this work, if the CPA of paired cluster centroids was no less than 0.85, the corresponding clusters were defined as shared synergies, the rest were mode-specific synergies.

### 2.7 Statistical analysis

To examine the significance of intra-mode similarity of muscle synergies under each condition (different training modes, different electrode configurations, and different movements), the intra-mode similarity metrics and their corresponding thresholds were included to perform the *t*-test. That is, the CPA and ED were used as the variables, respectively. For *t*-test, the chi-squared test was firstly used to examine if two samples were independent. And Wilcoxon rank-sum test, a non-parametric test was used to determine whether two independent samples were from populations having the same distribution. To examine if there is interaction effect of physical training mode and electrode configurate on the intra-mode similarity of muscle synergies, two-way ANOVA with Bonferroni correction was then conducted. The main effect of physical training mode and that of the electrode configurate were evaluated as well. On the other hand, the significance of inter-mode similarity of muscle synergies (different force-weight combinations) under each condition (different electrode configurations) was also examined by using *t*-test. In addition, to assess if there is significant difference between the intra-mode and inter-mode similarity of muscle synergies, paired *t*-test was separately performed in terms of CPA and ED values. In this work, *p*-value < 0.05 was taken as the threshold of statistical significance.

## 3 Results

### 3.1 High similarity of muscle synergies under specific training mode

**FIGURE 3:**
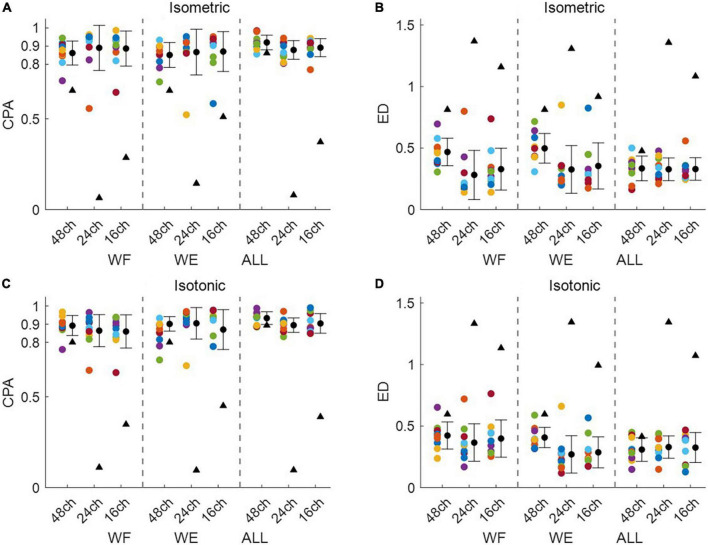
Intra-mode similarity of muscle synergies for wrist movements. Both **(A,B)** isometric mode and **(C,D)** isotonic mode were included, **(A,C)** CPA and **(B,D)** ED were used as the similarity metrics. Three different electrode configurations denoted as 48ch, 24ch, and 16ch were considered, respectively. Herein, circles with different colors are corresponding to different subjects, the black triangles denote CPA thresholds and ED thresholds under each condition.

Figure 3 shows intra-mode similarity of muscle synergies under each training mode for WE, WF, and combined dataset by WE and WF (ALL), where three electrode configurations were included for comparison. The force-pair-wise CPA, force-pair-wise ED, weight-pair-wise CPA, weight-pair-wise ED were calculated for each subject, and then averaged across all subjects under each condition. Figures 3A, B show that under isometric training mode, the averaged CPA values ranged from 0.85 to 0.92, all higher than the thresholds (denoted as black triangles), and the averaged ED values were all lower than the thresholds. Similar results were found for the isotonic mode (Figures 3C, D). Statistical analysis by using *t*-test indicates that the structure similarity of muscle synergies was significantly higher than the thresholds for each electrode configuration and each physical training mode (*p*-value < 0.05). We also examined whether physical training mode and electrode configurate affect the intra-mode similarity of muscle synergies by means of two-way ANOVA in terms of CPA and ED, respectively. It was found that neither physical training mode nor electrode configurate affect the intra-mode similarity of muscle synergies, and there was no interaction effect either.

**FIGURE 4:**
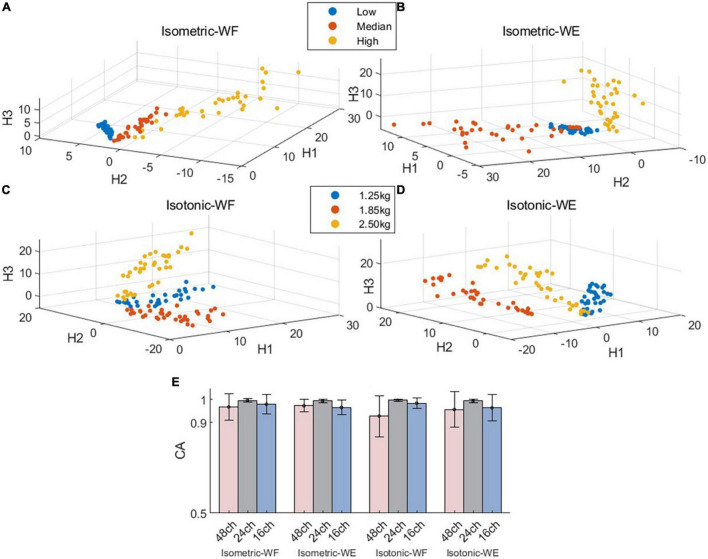
Spatial distribution of the mean values of activation coefficients and overall discrimination performance. **(A**–**D)** Demonstrate the mean value scatter plot of two wrist movements for Subject 01 under two training modes. **(E)** Classification accuracies for isometric WF, isometric WE, isotonic WF, and isotonic WE. Three different electrode configurations denoted as 48ch, 24ch, and 16ch were considered, respectively.

Next, we further investigated if the fixed intra-mode muscle synergies were modulated by activation coefficients to control wrist movements in different constrains. The CA to discriminate temporal activation coefficients corresponding to different force levels (or weights) was used as the performance metric. The scatter plots in Figures 4A–D demonstrate the mean values of activation coefficients, which were extracted from sliding windows of the first three principal components (denoted as H1, H2 and H3, respectively) for subject 01. It can be seen that scatters belonging to different force levels (or weights) have their own distinct boundaries, which implies that the activation coefficients corresponding to different force levels (or weights) could be discriminated well. The results in Figure 4E also confirm this hypothesis. The averaged CA corresponding to isometric WF, isometric WE, isotonic WF, isotonic WE were 96.75 ± 5.76%, 97.32 ± 2.70%, 92.75 ± 9.03%, and 95.66 ± 7.82% when using the 48-channel electrode configuration, which were slightly lower than that when using the other two electrode configurations, but still acceptable.

### 3.2 Decreased similarity of muscle synergies across different training modes

**FIGURE 5:**
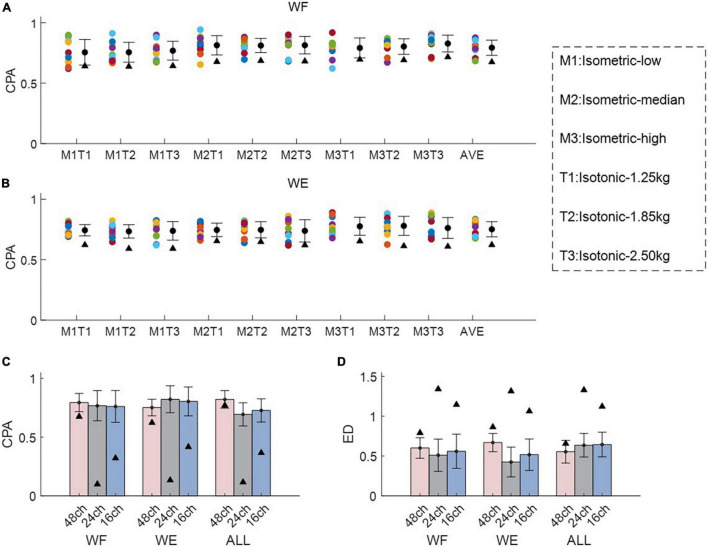
Inter-mode similarity of muscle synergies for wrist movements. **(A,B)** Demonstrate the inter-mode CPA for all force-weight combinations for WF and WE, where M1-M3 denote different force levels under isometric mode, and T1-T3 denote different weights under isotonic mode. Herein circles with different colors are corresponding to different subjects, the black triangles denote CPA thresholds and ED thresholds under each condition. In **(C,D)**, both CPA and ED were used as the similarity metrics, and three different electrode configurations denoted as 48ch, 24ch, and 16ch were considered, respectively. In **(A,B)**.

Figure 5 demonstrates the inter-mode similarity of muscle synergies between isometric mode and isotonic mode. For each motion with each electrode configuration, nine possible combinations of force-weight were separately analyzed and then averaged. Figures 5A, B illustrate the averaged CPA for each of the nine force-weight combinations when using the 48-channel electrode configuration, where WF and WE were analyzed, respectively. The results show that the averaged CPA values for all force-weight combinations were above 0.76 for WF, significantly higher than the thresholds according to the *t*-test (*p* < 0.05). But there was no significant difference among nine force-weight combinations (*p* > 0.05). Similar results were found for WE. These outcomes suggest that these paired force-weight training modes may share some common synergies even though they are different in kinematic performance.

The averaged inter-mode similarity of muscle synergies across all paired force-weight combinations for WF, WE, and the combined dataset was compared in Figures 5C, D, where CPA and ED were separately used, and three electrode configurations were included. It can be found that the 48- channel electrode configuration brought similar CPA values and ED values in comparison to the other two electrode configurations. Note when using the 48-channel electrode configuration, the averaged CPA were slightly higher than the threshold (denoted as black triangles), but the difference was significant according to the *t*-test (*p* < 0.05). While the other two electrode configurations brought obviously higher CPA and obviously lower ED in comparison to the thresholds (denoted as black triangles) (*p* < 0.05). These findings imply that despite the electrode configurations varies, there exist shared synergies between two training modes which have different kinematic performance.

Table 1 summarizes the averaged inter-mode similarity metrics and the averaged intra-mode similarity metrics across all subjects. Compared with the intra-mode similarity, the inter-mode CPA values decreased significantly (*p* < 0.05), and the inter-mode ED values increased significantly according to the paired *t*-test (*p* < 0.05). The attenuated similarity suggests that variation of physical training mode affects the modulation of muscle synergies.

**TABLE 1:** Comparison of intra-mode and Inter-mode similarity of muscle synergies.

Similarity metrics		Intra-mode						Inter-mode		
		Isometric			Isotonic					
Configuration		48ch	24ch	16ch	48ch	24ch	16ch	48ch	24ch	16ch
CPA	WF	0.86	0.89	0.89	0.89	0.86	0.86	0.79	0.77	0.76
	WE	0.85	0.87	0.87	0.90	0.90	0.87	0.75	0.82	0.80
	ALL	0.92	0.88	0.89	0.93	0.89	0.90	0.82	0.69	0.73
ED	WF	0.47	0.28	0.33	0.42	0.37	0.40	0.60	0.51	0.56
	WE	0.50	0.33	0.35	0.41	0.27	0.29	0.67	0.43	0.52
	ALL	0.33	0.33	0.33	0.31	0.33	0.33	0.55	0.64	0.65

**FIGURE 6:**
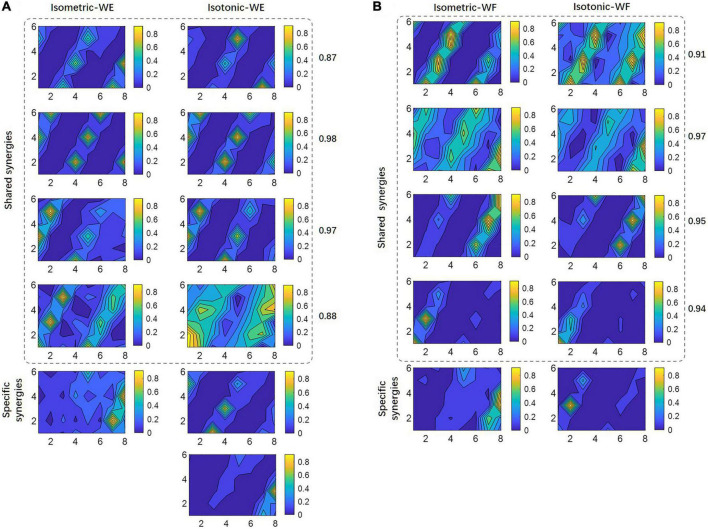
Shared synergies by two training modes and mode-specific synergies. For **(A)** WE and **(B)** WF, all synergy vectors after clustering are illustrated in 6×8 contour plots. Numbers denote the CPA between paired synergy vectors.

To further investigate the possible reason account for this result, shared muscle synergies belonging to two different training modes, as well as mode-specific synergies were extracted by means of clustering among all subjects. The results for WE were illustrated in Figure 6A, where isometric WE and isotonic WE have different numbers of synergy vectors, they have four shared synergies with similar spatial distributions, and the CPA values for paired synergy vectors were all no less than 0.85, while the mode-specific synergies show distinctly different spatial distributions. Similar results can be found for WF (Figure 6B). These mode-specific synergies may account for the attenuation of structure similarity.

## 4 Discussion

Toward clarifying the neuromuscular characteristics under isometric and isotonic training mode for wrist movements, we investigated the feasibility of applying HDEMG-based muscle synergy analyzing methods, and explored the modulation of muscle synergies under isometric training mode and isotonic training modes, respectively, as well as the inter-mode similarity of muscle synergies during wrist movements. This work may lead to an appropriate neuromuscular analysis method for motor function evaluation in clinical settings and provide valuable insights for the prescription of rehabilitation training therapies.

### 4.1 Robustness of applying HDEMG-based muscle synergy analysis

In this work, the HDEMG-based muscle synergy analyzing method was proposed to clarify the characteristics of muscle contraction for wrist movements under different training modes. The 48-channel electrode grid covered most of the superficial and deep muscles that control the hand and wrist movements. Unlike previous studies that utilized 10 or so EMG electrodes with specific muscle positioning (Singh et al., 2018), the HDEMG could provide more detailed spatial information. However, the robustness of applying HDEMG-based muscle synergy analysis has been rarely investigated. Therefore, in addition to the 48-channel monopolar electrode configuration, another two derived electrode configurations were also used for comparison in this work. It has been previously proved that the differential filter and the Laplace spatial filter could reduce the cross talk between adjacent EMG electrodes (Geng et al., 2014; Germer et al., 2021). Herein, we assumed that the HDEMG-based muscle synergy analyzing method would yield robust similarity of muscle synergies against different electrode configurations. If so, the feasibility of applying HDEMG-based muscle synergy analysis would be validated. Our results showed that both intra-mode and inter-mode similarity of muscle synergies were consistently robust to different electrode configurations, regardless of the similarity metrics (Figures 3, 5C, D). Moreover, in comparison to the other two electrode configurations, the 48-channel monopolar electrode configuration brought similar classification performance to discriminate the activation coefficients that corresponding to different force levels and different weights. These outcomes all suggest the robustness of applying HDEMG into muscle synergy analysis for wrist movements.

### 4.2 Modulation of muscle synergy under each training mode

Aiming at clarifying the modulation of muscle synergies for wrist movements, the intra-mode similarity of muscle synergies was computed. High intra-mode similarity was observed under variant levels of muscle contraction force in the isometric training mode, also under variant muscle tension in the isotonic training mode, independent of the similarity metrics (Figure 3). These findings confirm our initial hypothesis that there exist fixed muscle synergies representing the basic mechanism employed by the motor system. It is also in line with previous studies that established robust activation patterns of elbow and shoulder muscles during isometric force generation (Roh et al., 2012; Valk et al., 2019), suggesting that the CNS modulated activation of the same set of motor modules throughout specific training mode.

In addition to separate analysis for WF and WE, pooled HDEMG from WF and WE were also used to perform intra-mode muscle synergies extraction. Because for muscle synergy analysis, a very important assumption is that in every variant condition, there should be sufficient variability across trials for the identification of the EMG subspace underpinned by the muscle synergies that are potentially generalizable (d’Avella et al., 2003; Muceli et al., 2010; Valk et al., 2019). In other words, muscle synergy analysis relies critically on variability assumptions. Our results showed slightly higher similarity of intra-mode muscle synergies when using the combined dataset. It suggests that the muscle-contraction-force-variant synergies and muscle-tension-variant synergies are reflecting task constraints because of smaller amount of data variability fed into the algorithm, whereas the synergies from the combined dataset may reflect more neural constraints (d’Avella et al., 2003).

In presence of fixed muscle synergies across variant muscle contraction force and variant muscle tension, we further evaluated how the activation coefficient matrix modulates the excitation of specific synergy. Our results by means of pattern recognition displayed high classification accuracies (all above 92.75%) among all conditions (isometric WF, isotonic WF, isometric WE, isotonic WE) with different electrode configurations. It implies that there is an essential difference among the temporal coefficients corresponding to different force levels and muscle tension. The adaptive control strategy employed by the CNS to regulate the level of activation of muscle synergies by scaling the temporal coefficients may account for this outcome (Roh et al., 2012; Oscari et al., 2016; Israely et al., 2017; Zych et al., 2019).

### 4.3 Modulation of muscle synergy across two training modes

Another important issue we concerned was that if different training modes affect the modulation of muscle synergies. In this work, the inter-mode similarities of muscle synergies were found significantly lower than the intra-mode similarities (Table 1). These results suggest that the CNS may exploit different motor control strategies in response to different external constraints such as certain muscle contraction force or certain muscle tension during wrist motion. It is also in line with previous studies by examining the effect of body position with respect to gravity, movement size, and cranking mode on muscle coordination during arm cranking tasks (Botzheim et al., 2021). By further clustering analysis, most of the synergies were found shared between two training modes with similar spatial distribution, only a few synergies remained mode-specific (Figure 6). These findings imply that when facing high complexity of motor task, the CNS may have simplified the neural control at the expense of biomechanical efficiency (Valero-Cuevas et al., 2009; Roh et al., 2012; Pellegrino et al., 2020). Specifically, a fixed set of motor modules was used to achieve the targeted movement (four shared synergies for WE, for instance), while a few muscle synergies were recruited to keep certain muscle tension or certain muscle contraction force. In this study, it was harder to keep constant muscle tension in the sagittal plane than to keep constant muscle contraction force, which may account for that two mode-specific synergies the isotonic WE had while one mode-specific synergy the isometric WE had, and for their distinct spatial distributions. On the other hand, specific sensory feedback under different training mode might also contribute to the mode-specific synergies, because it has been proved that sensory feedback might adapt recruitment of muscle synergies to behavioral constraints, and the few specific synergies might represent afferent-specific modules or feedback reorganization of spinal neuronal networks (Cheung et al., 2005).

### 4.4 Limitations and future work

To summarize, the significance and unique of this work are from both scientific and engineering perspectives. Scientifically, we demonstrated that motor modularity hypothesis appears to hold even when HDEMGs are used. The results add to the growing evidence that muscle synergies are indeed the building blocks of movement construction. But our methodology and analysis have expanded the definition of synergies to include sub-muscle components that represent activities of subsets of motor units within a muscle (instead of whole-muscle components in the traditional definition of muscle synergies). Our analysis paves the way for future studies that aim to specifically investigate under what scenarios or behaviors sub-muscle components must be included in the definition of synergies to account for the observed EMG variability. For instance, the learning of very fine motor skills may involve plasticity of synergies that is only detectable at sub-muscle level. From the engineering/rehabilitation perspective, HDEMG synergy is another potential neurological marker that may precisely indicate the state of impairment of motor-impaired patients. Since HDEMG is more spatially precise, HDEMG synergies likely carry more diagnostic and prognostic values than traditional sEMG synergies.

However, there are also some limitations in this study. Firstly, only two fundamental strength training modes, i.e., the isometric exercise and isotonic exercise were considered. In clinical settings, isokinetic exercises are more often used for rehabilitation and recovery, and proved promising into promoting independent activation of muscle synergies in stroke survivors (Park et al., 2021). It has been recently involved to explore the physiological features of muscles when healthy subjects were performing elbow movements, where only eight sEMG electrodes were utilized (Sheng et al., 2022). According to our preliminary experiment, however, it was difficult to execute isokinetic wrist movements with the specialized machines that could generate consistent angular velocity and variable resistance, and avoid interference in a limited space in the meanwhile. Hence, in our future work, the experimental devices will be improved to facilitate the isokinetic wrist training for more comprehensive and systematic muscle synergy analysis.

Another limitation of this study is that only healthy individuals were recruited into the experiment, the obtained results cannot be extrapolated to patients with motor impaired patients directly. Because the group of disabled individuals with stroke, cerebral palsy, traumatic brain injury, and so on often display spasticity and abnormal synergy during strength training, yielding distinctly different muscle coordination patterns from that of the healthy subjects (Kong et al., 2019; Bekius et al., 2021; Li et al., 2021). Therefore, in our future for subjects with specific motor impairment, the modulation of muscle synergies needs to be analyzed independently.

Thirdly, muscle synergy has been mostly used to quantify the activation of multiple muscles to achieve a goal efficiently in previous literatures (d’Avella et al., 2003; Muceli et al., 2010; Torres-Oviedo and Ting, 2010; Israely et al., 2017; Pellegrino et al., 2020). With the HDEMG electrode grid design in this work, however, it became hard to quantify the biomechanical contribution of each synergy vector, especially when there was no anatomical localization information of each HDEMG electrode. It is also hard to infer the neuromechanical or neurophysiological basis of wrist movements further. This work was mostly extensive on computational aspects rather than physiological ones. In our future work focused on the patients with neurological disorders, the abnormal modulation of muscle synergies in terms of biomechanical properties will be elaborated with an attempt to provide new physiological insights into synergies.

Lastly, it’s noteworthy that the muscle coordination could only reveal the general mechanism underlying movement generation. To clarify how the activation coefficients of muscle synergy correlate with the recruited motor units and firing rate may provide deep understanding of the neuromuscular coupling mechanisms, and offer essential insights for the development of neurorehabilitation strategies. For this concern, HDEMG-based motor unit decomposition techniques will be employed in the future work. Note the HDEMG grid deployed in this study had a diameter of 5 mm, and the IED was around 2 cm, which was depended on the forearm size of each subject. Strictly speaking, it was multichannel EMG with compact and dense distribution, rather than the HDEMG grid that often used for motor unit decomposition.

## Data availability statement

The data that support the findings of this study are available upon reasonable request from the authors.

## Ethics statement

The studies involving human participants were reviewed and approved by the Research Ethics Board of Shenzhen Institute of Advanced Technology, Chinese Academy of Sciences. The patients/participants provided their written informed consent to participate in this study.

## Author contributions

YG, ZC, and YZ designed the experiment. VC provided technical consulting. YG and ZC performed the experiments, analyzed the data, and drafted the manuscript. YG, ZC, YZ, and VC interpreted the data. YG, ZC, YZ, VC, and GL edited the manuscript. All authors contributed to the article and approved the submitted version.
